# Ocular sarcoidosis and tuberculous lymphadenopathy: coincidence or real association

**DOI:** 10.1007/s12348-011-0021-2

**Published:** 2011-02-26

**Authors:** Jiun-Yo Lin, Shwu-Jiuan Sheu

**Affiliations:** 1 Department of Ophthalmology, Kaohsiung Veterans General Hospital, 386, Ta-Chung 1st Road, Kaohsiung, 813 Taiwan; 2 School of Medicine, National Yang-Ming University, Taipei, Taiwan

## Abstract

Tuberculosis and sarcoidosis share similarity in histopathologic findings and clinically occur in association with each other occasionally. Tuberculosis should always be ruled out before the diagnosis of sarcoidosis. But, the diagnosis is often complicated, especially in extrapulmonary cases. Here we present a case of bilateral vitreous hemorrhage with uveitis. Ocular sarcoidosis was initially diagnosed based on the characteristic ocular findings, negative results on chest radiography, tuberculosis culture, and polymerase chain reaction of aqueous, as well as simultaneous presence of panda and lambda sign on gallium-67 scans. The ocular condition improved after pars plana vitrectomy and systemic steroid therapy. However, TB lymphadenopathy but no recurrent ocular inflammation was found 6 years later. The patient received anti-TB treatment for 6 months thereafter. The eyes remained silent except cataract progression and glaucoma under two medications during this period. In conclusion, TB could occur coincidently or in association with sarcoidosis, continued follow-up is important for patients with ocular sarcoidosis.

## Introduction

Sarcoidosis is a multisystemic, granulomatous disease of unknown etiology that may affect any organ system. It is characterized by the accumulation of T lymphocytes and mononuclear phagocytes with the formation of noncaseating epithelioid granulomas in affected organs [1]. The trigger for this T cell and macrophage interaction is still uncertain, although bacterial, viral, and environmental antigens have all been studied. Recent literatures renewed the interest in mycobacteria as a causative agent in sarcoidosis tissue [2, 3]. Here we present a case of presumed ocular sarcoidosis diagnosed by ocular manifestation and treatment course, who developed tuberculous lymphadenopathy 6 years later without recurrence of ocular inflammation.

## Case report

**Fig. 1:**
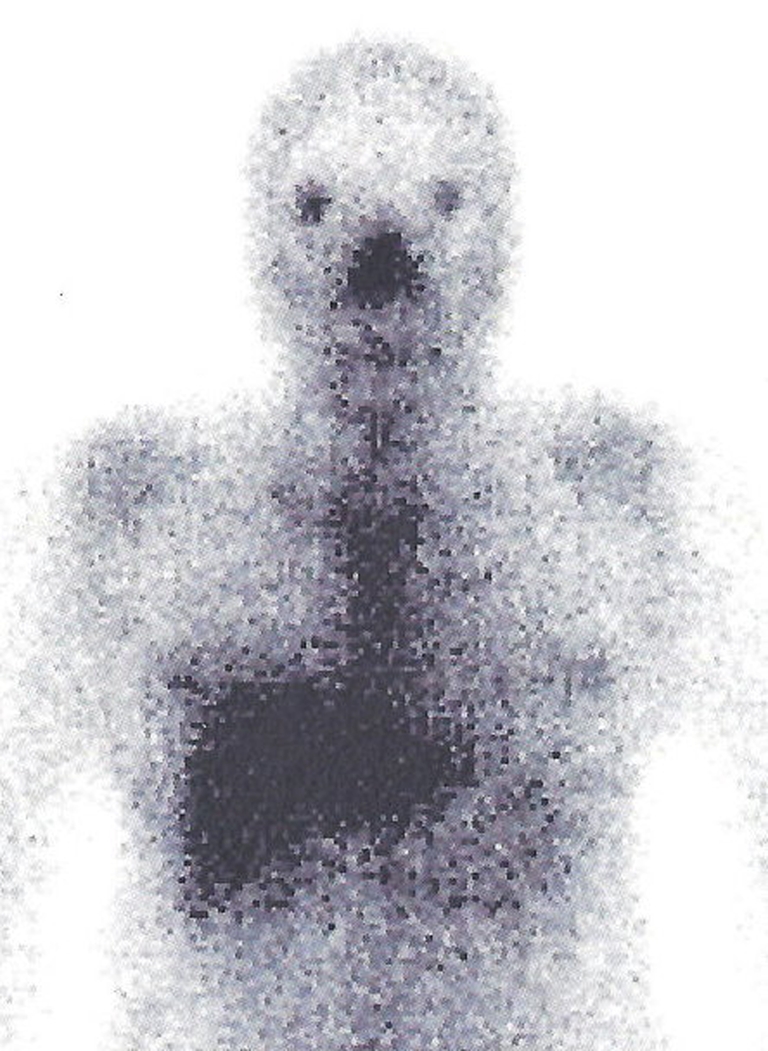
Gallium-67 scans showed panda sign of increased uptake in lacrimal glands and lambda sign of lymph nodes in the mediastinum

**Fig. 2:**
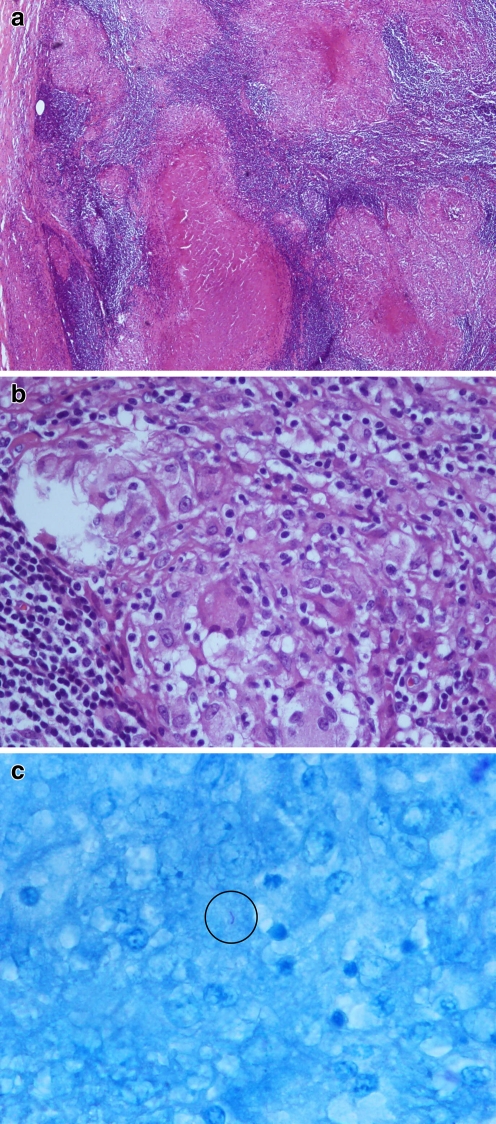
Pathological finding from lymph node biopsy showed granuloma with caseous necrosis (hematoxylin and eosin, ×40) (**a**), Langhans giant cell (hematoxylin and eosin, ×400) (**b**), as well as acid-fast bacilli (Kinyoun's crystal Fuchsin acid-fast stain, ×1,000) (**c**)

**Fig. 3:**
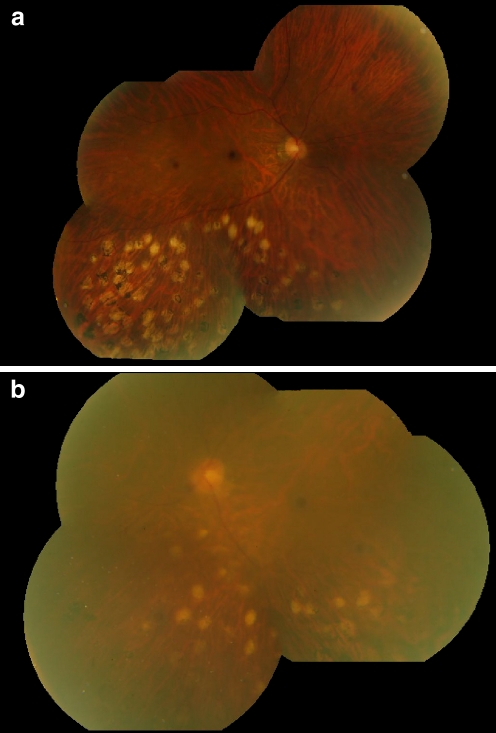
Postoperative color fundus picture of both eyes showed sheathing vessels and scattered laser scars at the inferior retina. **a** Right eye. **b** Left eye

A 35-year-old female was referred to our department with severe visual impairment for months. She was quite healthy until 1 year ago when she began to suffer recurrent episodes of uveitis with mutton fat keratic precipitates (KPs) in both eyes. On presentation, visual acuity was counting finger in right eye and hand motion in left eye. Ocular examination showed mild anterior uveitis with mutton fat KPs and dense vitreous hemorrhage in both eyes. Systemic workup including general physical examination, complete blood counting, differential classification, blood biochemical tests, rheumatic factor, antinuclear antibody, human immunodeficiency virus, venereal disease research laboratory test, anticardiolipin antibody, and chest radiography were within normal ranges. Due to persistent vitreous hemorrhage and disabled vision, pars plana vitrectomy was performed in the left eye and then the right eye 2 months later. Sign of peripheral vasculitis with nodular choroid infiltration was noted during operation in both eyes. Aqueous for TB culture and polymerase chain reaction (PCR) was negative. Angiotensin-converting enzyme was not done due to the unavailability in our hospital. Gallium-67 scans showed panda sign of increased uptake in lacrimal glands and lambda sign of lymph nodes in the mediastinum (Fig. 1). Chest computed tomography (CT) showed a small ground glass opacity nodule in the subpleural space of the right lower lobe, more in favor of inflammatory process. The patient refused further biopsy for histopathological study. Gonioscopy showed trabecular meshwork nodules and tent-shaped peripheral anterior synechiae. Based on these findings, ocular sarcoidosis was impressed and systemic and topical steroids administered in a tapering dose over 6 months. The eyes remained silent with controllable glaucoma in both eyes after surgery. Unfortunately, painless enlargement of lymph node at her right neck was noted 6 years later. CT showed multiple large lymph nodes over bilateral lower neck. Gallium-67 scans showed increase uptake in bilateral lacrimal glands. Lymph node biopsy showed granuloma with caseous necrosis, Langhans giant cell, as well as acid-fast bacilli (Fig. 2). Repeated chest radiography was negative. Sputum was negative for tuberculosis by culture and acid-fast stain. Three combined antituberculosis medications (rifampin, isoniazid, and pyrazinamide) were given for 6 months. The eyes remained silent except cataract progression and glaucoma under two medications during this period (Fig. 3).

## Discussion

The gold standard for diagnosis of sarcoidosis is histopathologic examination. Considering the fact that ocular sarcoidosis may occur in the absence of apparent systemic involvement, and the fact that biopsy is usually reluctantly accepted by uveitis patients, the International Workshop on Ocular Sarcoidosis (IWOS) established an international criteria for the diagnosis of ocular sarcoidosis [4]. The consensus identified seven clinical signs suggesting ocular sarcoidosis. Although there was no pathologic proof of sarcoidosis in our case, her ocular manifestations met at least five of the IWOS criteria. Though the lacrimal gland uptake of gallium (panda sign) is not a specific sign for sarcoidosis, her simultaneous presence of lambda and panda sign on Ga-67 scans gave some support to sarcoidosis [5]. As guided, other granulomatous diseases, including tuberculosis, foreign body reaction, bacterial and viral infections, should be excluded before the diagnosis of sarcoidosis. TB culture and PCR for aqueous were negative. Other laboratory and image study ruled out the other possible etiologies. Besides, the ocular inflammation subsided after pars plana vitrectomy and systemic corticosteroid. There was no recurrence of inflammation even at the time of tuberculous lymphadenopathy. Presumed ocular sarcoidosis was impressed based on the ocular manifestations and treatment course. We could not make any conclusion whether ocular sarcoidosis and TB lymphadenopathy in this patient is just coincident or in real association with each other.

Despite the increasing understanding of the immune responses behind the formation and maintenance of the granulomatous process in sarcoidosis, its etiology remained elusive. Mycobacteria have been implicated as a cause of sarcoidosis, although the demonstration of mycobacterial DNA was inconsistent in different reports [6, 7]. Recent studies by detecting *Mycobacterium tuberculosis* catalase–peroxidase have renewed interest in mycobacteria as a causative agent in sarcoidosis [2, 3]. In our case, TB was ruled initially due to negative results of chest X-ray, aqueous TB culture, and PCR. Tuberculin skin test was not done as our CDC did not identify the significance of PPD in adult who received routine bacille Calmette–Guerin vaccination. The ocular inflammation revolved after systemic steroid, and remained silent even at the presence of TB lymphadenopathy. Our speculation is that the presumed ocular sarcoidosis might be triggered by TB antigen in extraocular infection, which reactivated and presented as TB lymphadenopathy 6 years later.

## Conclusion

TB could occur coincidently or in association with sarcoidosis, continued follow-up is important for patients with ocular sarcoidosis, even in silent case.
